# *Cryptococcus neoformans *meningitis in a diabetic patient - the perils of an overzealous immune response: a case report

**DOI:** 10.1186/1757-1626-2-209

**Published:** 2009-11-19

**Authors:** Anurag Kushawaha, Neville Mobarakai, Nirupama Parikh, Alex Beylinson

**Affiliations:** 1 Department of Medicine, Staten Island University Hospital, Staten Island (NYC), USA; 2 Division of Infectious Diseases, Staten Island University Hospital, Staten Island (NYC), USA; 3 Division of Hospitalists, Staten Island University Hospital, Staten Island (NYC), USA

## Abstract

Uncontrolled diabetics are prone to infections due to numerous factors as the glucose-rich blood serves as an excellent media for growth. Cryptococcus neoformans is an opportunistic fungus that is an important cause of CNS infections among immunocompromised patients, but it has only sporadically been reported in non-HIV-positive persons. The presence of elevated pro-inflammatory cytokines and abnormalities in numerous systemic indicators of inflammation in diabetics makes it conceivable that diabetics mount an exaggerated immune response to C. neoformans (paradoxical to their defective immune state) leading to grave outcomes. We present a fatal case of C. neoformans meningitis in a diabetic patient which emphasizes the perils of an overzealous immune response.

## Case presentation

A 72-year-old Caucasian male of Italian-American descent was admitted for unremitting, severe, right frontal headache, gait ataxia, and slurred speech for the preceding five days. His past medical history was notable for insulin-dependent diabetes, hypertension, and mitral valve replacement. Social history was unremarkable; he was a retired shipyard laborer. The patient's wife denied recent travel or pets.

The history goes back ten days prior to presentation when the patient was evaluated at an outside institution for persistent headaches and gait ataxia. At that time, computed tomography of the head was negative for an acute bleed or mass. The patient was presumed to have metabolic encephalopathy and was admitted to the rehabilitation ward for further evaluation of the gait disturbance. He was transferred back to the medical ward for change in mental status and worsening encephalopathy.

Initial vital signs were notable for a maximum temperature of 101.2°F, BP 120/70 mmHg, pulse 78 beats/min., respiratory rate 20 breaths/min. Physical examination revealed a lethargic man, but who was arousable to verbal and tactile stimuli; he moved all extremities. Reflexes were intact and he followed simple commands. The remainder of the physical exam was normal.

Admission labs were notable for glucose of 225 mg/dL, creatinine of 1.4 mg/dL, ammonia level of 58 umol/L, and WBC count 14.3 × 10^3^cells/mm^3 ^(81% neutrophils, 10% lymphocytes). Other routine labs were unremarkable. The urinalysis showed moderate bacteruria and blood with 12-20 WBCs/hpf.

Computed tomography of the head, chest radiograph, and EKG were unremarkable. Renal ultrasound revealed normal kidneys with no evidence for hydronephrosis. Blood and urine cultures were drawn; the patient was started on intravenous ciprofloxacin, and was admitted for change in mental status secondary to urosepsis.

**Figure 1:**
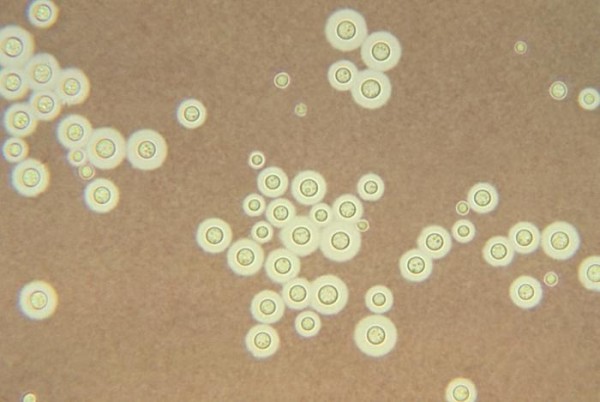
**India ink preparation of cerebrospinal fluid from a patient with cryptococcal meningitis showing the budding yeast cells of *C. neoformans *surrounded by a characteristic wide gelatinous capsule**. The yeasts also show narrow-base budding and characteristic variation in size.

Over the next 48 hours, the patient began spiking fever above 101°F and became non-verbal. A lumbar puncture was conducted and revealed clear, colorless CSF, with 150 WBC/mm^3 ^(95% lymphocytes, 5% neutrophils), 0 RBC/mm^3^, protein 158 mg/dL, glucose 73 mg/dL (serum level, 380), chloride 121 mEq/L. Opening pressure of the CSF was not measured. The CSF gram stain showed the presence of budding yeast and India ink stain was positive (See figure 1). The patient was started on intravenous amphotercin B and flucytosine.

Over the next few days, the patient became more responsive and mild improvement in mental status was noted. However, there was no improvement in his speech. During this time, his serum glucose remained elevated, ranging between 274 mg/dL to 364 mg/dL. The amphotericin B was changed to a liposomal formulation because of renal insufficiency. Subsequent CSF cytology revealed organisms consistent with *Cryptococcus neoformans*. The CSF culture was also positive for *C. neoformans*. The CSF cryptococcal antigen titer using latex particle agglutination was over 1:1024. Further testing for Lyme antibodies and HIV antibodies was negative. An echocardiogram revealed normal left ventricular size and function with no vegetations. Blood and urine cultures were negative. The antifungals were continued.

By hospital day 10, the patient became more arousable to verbal and tactile stimuli and would answer simple questions. However, he remained lethargic and febrile. The WBC count remained elevated at 13.9 × 10^3^/mm^3^, with 89% granulocytes. The serum glucose was 263 mg/dL and continued to be uncontrolled. A repeat CT head showed the new development of moderately enlarged third and lateral ventricles consistent with mild communicating hydrocephalus. A repeat lumbar puncture was recommended to assess the CSF opening pressure. While the LP was being attempted, the patient became cyanotic and unresponsive. The patient was emergently intubated and advanced cardiac life support was started. The initial cardiac rhythm was asystole, which with ACLS became a wide complex bradycardia, then subsequently ventricular fibrillation and finally refractory asystole. Despite aggressive attempts at resuscitation, the patient expired.

An autopsy was conducted. The CNS examination revealed findings consistent with cryptococcal meningitis affecting the basal leptomeninges, which were opaque and thickened. Furthermore, there was reactive connective tissue obstructing the outflow of CSF consistent with hydrocephalus. There was marked cerebral edema with uncal grooving and midbrain compression. Sections of the brain showed numerous areas of tissue destruction and gelatinous, mucoid material within the subarachnoid space. The meningeal exudate consisted of chronic inflammatory cells along with cryptococci. Some sections of the brain also disclosed macrophages and multinucleated giant cells indicating granulomatous inflammation. The cause of death was herniation of the cerebellar tonsils through the foramen magnum with subsequent impaction and acute compression of the medulla oblongata causing respiratory and circulatory arrest. There was no evidence of acute myocardial infarction or pulmonary embolism.

## Discussion

*Cryptococcus neoformans *is an opportunistic fungus that is an important cause of CNS infections among immunocompromised patients, but it has only sporadically been reported in non-HIV-positive persons. The most common forms of immunosuppression (other than HIV) include chronic glucocorticoid use, history of organ transplantation, malignancy, as well as sarcoidosis and liver failure. During a 17-year, retrospective review study of non-HIV patients with cryptococcal infection, Kiertiburanakul et al showed that the most common associated conditions included immunosuppressive drug treatment (41%), presence of systemic lupus erythematosus (16%), malignancies (16%), and diabetes mellitus (14%) [1]. Our case represents this small, but significant, fraction of diabetic patients.

*Cryptococcus neoformans *produces infection following inhalation through the respiratory tract. The organism then disseminates hematogenously and has a propensity to infect the CNS (neurotropism). This predilection has been postulated to be due to several factors: the high level of dopamine in the CNS potentially serves as a reservoir of melanin production which may increase fungal virulence [2]; the CSF provides a rich medium to promote fungal growth; and the local production of mannitol by the fungus may contribute to cerebral edema and interfere with phagocyte function [3].

Our patient was diabetic and succumbed to CNS cryptococcosis. During the hospitalization, his blood sugar was consistently elevated. Uncontrolled diabetics are much more prone to infections due to numerous factors as the glucose-rich blood serves as an excellent media for growth and when infections do occur, they are more serious. Diabetic ketoacidosis, for example, is caused by or complicated by an infection in many cases. Some of the commonly seen infections seen in diabetics, including rhinocerebral mucormycosis, malignant otitis externa, emphysematous cholecystitis/pyelonephritis, and acute necrotizing fasciitis reflect defects of the immune system. Hyperglycemia is known to cause impaired polymorphonuclear chemotaxis, abnormal phagocytosis, and deficient cell-mediated immunity. The most important host response to cryptococcal infection is most probably cell-mediated immunity as the effector cells against the yeast include CD4+ and CD8+ lymphocytes, natural killer cells, and activated phagocytes that produce granulomatous inflammation [4]. Granuloma formation also requires the presence of TNF, IL-2, IL-12, and inflammatory cytokines, such as monocyte chemotactic protein-1(MCP-1), and macrophage inflammatory protein-α (MIP-α), which collectively are involved in recruiting host cells to the site of infection leading to inflammation [5]. Since serum levels of MCP-1 are elevated in patients with type 2 diabetes [6], as are levels of TNF-α and interleukin-6 [7], it is conceivable that diabetics mount an exaggerated immune response to *C. neoformans *(paradoxical to their defective immune state) leading to fatal outcomes. Furthermore, subclinical systemic inflammation and abnormalities in numerous systemic indicators of inflammation have been reported in type 2 diabetics. Increased resting systemic inflammation and elevated basal cytokine formation may in part be due to advanced glycation end products (AGEs). An increased formation of AGEs occurs in poorly controlled diabetics, which could play a role in increased basal cytokine production [8].

This suggests that anti-inflammatory measures may have an adjunctive role in the therapy of cryptococcal meningitis in diabetic patients. Corticosteroids decrease vascular permeability, inhibit leukocyte migration to sites of inflammation, and intracellularly block the transcription of pro-inflammatory proteins. Using anti-inflammatory medications to help treat infectious diseases has been described. For patients with pneumococcal meningitis, tuberculous meningitis, tuberculous pericarditis, severe typhoid fever, tetanus, or pneumocystis pneumonia with moderate to severe hypoxemia, treatment with corticosteroids has been shown to improve survival [9]. Though it remains controversial, case reports have been described regarding the beneficial use of corticosteroids in non-HIV patients with cryptococcal meningitis [10].

In conclusion, our case of overwhelming cryptococcal meningitis in a diabetic patient demonstrates the perils of an intense inflammatory response, which may play a crucial role in the pathogenesis of CNS cryptococcal disease in this patient population. The implications of this case are noteworthy. Cryptococcal meningitis should be considered in the differential diagnosis for all persons, including non-HIV patients, who present with chronic headache, especially if there is also the presence of fever, neurologic deficits, or neuroradiologic findings. The neurotropic property of *C. neoformans *and its numerous virulence factors add to its lethality. Diabetic patients are at increased risk for infections due to numerous defects in their immune system and immune response. Uncontrolled hyperglycemia has been shown to cause alterations in host defense and, thereby, increased susceptibility to infections. Pro-inflammatory cytokines, such as TNF-α and various interleukins, are elevated in hyperglycemic states and are significantly lowered by insulin therapy. Since immune cell dysfunction and desynchronized inflammatory cytokines may be partially responsible for the greater risk of infection and the corresponding high morbidity and mortality in diabetic patients, agents capable of altering host cell function (i.e., exogenous anti-cytokine therapy), potentially hold an attractive therapeutic modality to supplement current antimicrobial agents in the future.

## Consent

The patient's next of kins' consent was obtained prior to composing this manuscript. A copy of the written consent is available for review from the journal's Editor-in-Chief.

## Competing interests

The authors disclose they have no proprietary interest or additional financial support for publication of this article.

## Authors' contributions

All authors have read and approved the final manuscript. AK was the major contributor of the manuscript, conducted literature review, and was involved in manuscript revisions. NM was involved in direct patient care as the infectious disease consultant and was involved in manuscript revisions. NP was involved in direct patient care as the hospitalist and was involved in manuscript revisions. AB was involved in direct patient care as the attending physician.

## References

[B1] Kiertiburanakul S Wirojtananugoon S Pracharktam R Sungkanuparph S Cryptococcosis in human immunodeficiency virus-negative patients Intl Journal of Infect Dis 2006 10 1 72 78 10.1016/j.ijid.2004.12.004 16288998

[B2] Kwon-Chung KJ Rhodes JC Encapsulation and melanin formation as indicators of virulence in Cryptococcus neoformans Infect Immun 1986 51 1 218 23 3079732 10.1128/iai.51.1.218-223.1986 PMC261090

[B3] Wong B Perfect JR Beggs S Wright KA Production of the hexitol D-mannitol by Cryptococcus neoformans in vitro and in rabbits with experimental meningitis Infect Immun 1990 58 6 1664 70 2111284 10.1128/iai.58.6.1664-1670.1990 PMC258702

[B4] Huffnagle GB Lipscomb MF Lovchik JA Hoag KA Street NE The role of CD4+ and CD8+ T cells in the protective inflammatory response to a pulmonary cryptococcal infection J Leukoc Biol 1998 55 35 42 10.1002/jlb.55.1.35 7904293

[B5] Aguirre K Havell EA Gibson GW Johnson LL Role of tumor necrosis factor and gamma interferon in acquired resistance to Cryptococcus neoformans in the central nervous system of mice Infect Immun 1995 63 1725 1731 7729878 10.1128/iai.63.5.1725-1731.1995 PMC173216

[B6] Nakamura K Yamagishi S Adachi H Circulating advanced glycation end products (AGEs) and soluble form of receptor for AGEs (sRAGE) are independent determinants of serum monocyte chemoattractant protein-1 (MCP-1) levels in patients with type 2 diabetes Diabetes/Metabolism Research and Reviews 2007 24 2 109 114 10.1002/dmrr.766 17694504

[B7] Charo IF Ransohoff RM The Many Roles of Chemokines and Chemokine Receptors in Inflammation N Engl J Med 2006 354 610 621 10.1056/NEJMra052723 16467548

[B8] Stehouwer C Lambert J Donker A van Hinsbergh VWM Endothelial dysfunction an pathogenesis of diabetic angiopathy Cardiovasc Res 1997 34 55 68 10.1016/S0008-6363(96)00272-6 9217873

[B9] McGee S Hirschmann J Use of Corticosteroids in Treating Infectious Diseases Arch Intern Med 2008 168 10 1034 1046 10.1001/archinte.168.10.1034 18504331

[B10] Lane M Mcbride J Archer J Steroid responsive late deterioration in *Cryptococcus neoformans *variety *gattii *meningitis Neurology 2004 63 713 14 15326249 10.1212/01.wnl.0000134677.29120.62

